# Perceived Organisational Support, Work Engagement and Disaster Literacy Among Critical Care Reserve Nurses: A Cross‐Sectional Path Analysis

**DOI:** 10.1155/jonm/2921358

**Published:** 2026-11-06

**Authors:** Chang Shu, Zhijun Shu, Ziyan Yu, Lilan Wang, Shaohua Hu, Bingbing Hu

**Affiliations:** ^1^ School of Nursing Anhui Medical University Hefei Anhui China ahmu.edu.cn; ^2^ Nursing Department The First Affiliated Hospital of Anhui Medical University Hefei Anhui China ahmu.edu.cn; ^3^ Department of Neurosurgery The First Affiliated Hospital of Anhui Medical University Hefei Anhui China ahmu.edu.cn

**Keywords:** critical care nursing, disaster literacy, disaster nursing, perceived organisational support, work engagement

## Abstract

**Aim:**

To examine the associations among perceived organisational support, work engagement and self‐reported disaster literacy in critical care reserve nurses, and to assess the indirect association between perceived organisational support and self‐reported disaster literacy through work engagement.

**Background:**

Hospital resilience during public health emergencies depends on a reserve nursing workforce that can be rapidly mobilised and can translate disaster‐related information into safe clinical action. However, the organisational and motivational conditions associated with disaster literacy in this workforce remain insufficiently understood.

**Methods:**

This cross‐sectional survey included all 113 nurses who were enrolled in a critical care training programme and registered in an emergency reserve pool at a tertiary hospital in China. Data from validated self‐report instruments were analysed using Pearson correlations and observed‐variable path analysis with 5000 bootstrap resamples. The adjusted model included demographic and training covariates.

**Results:**

Perceived organisational support correlated with work engagement (*r* = 0.588) and self‐reported disaster literacy (*r* = 0.540), and work engagement correlated with self‐reported disaster literacy (*r* = 0.565). In the adjusted model, perceived organisational support was associated with work engagement (*β* = 0.570) and self‐reported disaster literacy (*β* = 0.307), while work engagement was associated with self‐reported disaster literacy (*β* = 0.449). The standardised indirect association through work engagement was *β* = 0.256 (bias‐corrected 95% CI [0.135, 0.440]).The indirect and direct estimates represented approximately 45.5% and 54.5%, respectively, of the total model‐estimated association; these percentages reflect mathematical coefficient decomposition in the specified cross‐sectional model and should not be interpreted as proportions of a causal mediated effect.

**Conclusions:**

Perceived organisational support, work engagement and self‐reported disaster literacy were positively associated; however, the cross‐sectional design precludes conclusions about temporal order or causality.

**Implications for Nursing Management:**

Nurse managers should consider organisational support and work engagement alongside technical competence when developing reserve‐workforce preparedness programmes. Strategies such as protected training time and psychologically safe debriefing warrant prospective evaluation.

## 1. Introduction

Public health emergencies and disasters can disrupt hospital operations, increase demand for critical care and require nurses to work in uncertain, resource‐constrained environments. Hospital preparedness therefore depends not only on emergency plans and equipment but also on a nursing workforce able to interpret evolving information, coordinate with multidisciplinary teams and translate guidance into safe clinical action. In the participating hospital, critical care reserve nurses were registered in an emergency reserve pool and enrolled in a structured critical care skills training programme. Because these nurses may be mobilised when additional critical care capacity is required, ensuring their readiness is a nursing‐management responsibility as well as a matter of clinical competence. Disaster literacy (DL) refers to the ability to access, understand, appraise and apply disaster‐related information across prevention, preparedness, response and recovery [1]. In nursing, it also includes communication, teamwork, emotional regulation, leadership, information appraisal and adaptation under uncertainty [2, 3]. This broader understanding is reflected in disaster‐nursing competency frameworks issued by the International Council of Nurses (ICN), alone or in collaboration with the World Health Organisation, in 2009, 2019 and 2022 [4–6].

Despite these frameworks, reviews continue to identify gaps in nurses’ practical competence, disaster preparedness and psychological readiness [7, 8]. Recent meta‐analytic evidence indicates that overall preparedness remains moderate, with disaster skills and management abilities requiring further strengthening [9]. Chinese studies have also reported variation in preparedness and DL according to region, hospital level, previous training and disaster‐response experience [10, 11]. Structured emergency training may improve competence and professional value [2, 12], and theory‐informed programmes integrating conceptual, practical and evidence‐based content are recommended [13]. However, training alone may not ensure sustained readiness, which requires ongoing participation in preparedness activities, reflection on practice and application of learning. The Job Demands–Resources (JD–R) model provides a framework for examining these conditions, proposing that job resources support motivation and favourable work outcomes [14, 15]. Perceived organisational support (POS), defined as employees’ belief that the organisation values their contribution and cares about their well‐being [16, 17], can function as such a resource. Previous studies have linked POS with work engagement (WE) and organisational citizenship behaviour among hospital nurses [18], while research among Chinese nurses has reported positive associations between organisational support and engagement [19, 20]. Visible management practices may also signal that preparedness is an organisational priority rather than an additional individual obligation [21].

WE, characterised by vigour, dedication and absorption [22], may help explain how nurses use organisational resources. Disaster preparedness requires nurses to seek information, practise skills, participate in simulations, reflect on performance and integrate new knowledge into clinical judgement. More engaged nurses may therefore participate more actively in preparedness activities. However, evidence remains limited regarding how organisational support and WE are jointly associated with DL among hospital reserve nurses. Addressing this gap is relevant to nursing management because managers can influence both the availability of organisational resources and how visible these resources are to nurses. The present study therefore examined the associations among POS, WE and self‐reported DL in critical care reserve nurses. Based on the JD–R model, we proposed four theory‐informed hypotheses: H1, POS would be positively associated with WE; H2, WE would be positively associated with self‐reported DL; H3, POS would be positively associated with self‐reported DL; and H4, the observed data would be consistent with an indirect association between POS and self‐reported DL through WE. Because all variables were measured at one time point, these hypotheses concern statistical associations rather than temporal or causal effects.

## 2. Methods

### 2.1. Design and Setting

Following ethical approval, a cross‐sectional survey was conducted between March and April 2026 in a tertiary general hospital in Hefei, Anhui Province, China. Data were collected at baseline, immediately before the start of a six month critical care skills training programme. The study examined the associations among POS, WE and DL in critical care reserve nurses. Because all variables were measured concurrently, temporal relationships could not be established. The study is reported in accordance with the STROBE guideline for cross‐sectional studies. The name of the participating hospital was withheld for blinded review.

### 2.2. Participants and Recruitment

The target population comprised all 113 nurses registered in the hospital’s critical care skills training programme and emergency reserve pool during the study period. All eligible nurses were invited to participate, and all provided written informed consent and completed the questionnaire. To reduce potential common method variance, questionnaires were completed anonymously and independently. Participants were informed that there were no right or wrong answers and that their responses would be used for research purposes only. Standardised instructions were used during questionnaire administration. Therefore, 113 questionnaires were included in the analysis.

Eligible participants were active nursing personnel registered in both the critical care skills training programme and the emergency reserve pool. Nurses were excluded if they were not enrolled in the programme or did not complete the survey because of resignation, leave, or withdrawal. No inferential subgroup comparisons were performed because several groups were small or markedly unequal in size, and demographic comparisons were not the primary study objective.

Because all 113 eligible nurses participated, the study included the entire defined reserve workforce rather than a probability sample.

### 2.3. Measures

#### 2.3.1. Participant Characteristics

A study‐specific questionnaire collected information on gender, age, ethnicity, education, marital status, professional title, whether a disaster‐response team had been established, and previous school‐based disaster education and on‐the‐job disaster training. These variables were selected to characterise the reserve workforce and to reflect background factors previously discussed in disaster‐preparedness research [7, 9]. The distributions also provide information about the homogeneity of the sample and the extent to which particular categories were sparsely represented.

#### 2.3.2. POS

The Chinese POS Scale adapted for nurses was used [23]. The 13‐item instrument comprises emotional support (10 items) and instrumental support (three items). The nursing adaptation was developed from an existing Chinese organisational support scale and refined through nurse interviews, pretesting and expert review. The original validation supported its content validity through expert assessment and correlations between the subscales and total scale. Items are rated from 1 (strongly disagree) to 5 (strongly agree), producing total scores from 13 to 65, with higher scores indicating greater POS. In the present sample, Cronbach’s *α* was 0.979.

#### 2.3.3. WE

The 17‐item Utrecht WE Scale (UWES) developed by Schaufeli et al. [22] was used. It assesses vigour (six items), dedication (five items) and absorption (six items). A Chinese adaptation study supported the three‐factor structure of WE and demonstrated expected associations with health status, coping strategies and job characteristics, although two low‐discrimination items were removed during the Chinese validation process [24]. Items are rated from 0 (never) to 6 (always), with total scores ranging from 0 to 102. Higher scores indicate greater WE. Cronbach’s *α* was 0.972 in the present sample.

#### 2.3.4. DL

The Clinical Nurses’ DL Self‐Assessment Questionnaire was developed and validated in China [2]. It comprises 34 items across three dimensions: functional literacy (12 items), interactive literacy (nine items) and critical literacy (13 items). During instrument development, exploratory factor analysis supported the three‐factor structure; the Kaiser–Meyer–Olkin (KMO) measure was 0.983, and Bartlett’s test was significant (*p* < 0.001), with the three factors explaining 79.98% of the total variance. Item‐level content validity indices ranged from 0.80 to 1.00, and the scale‐level content validity index was 0.823. Confirmatory validation further showed standardised factor loadings above 0.60, average variance extracted values above 0.50 and composite reliability values above 0.90, supporting convergent and discriminant validity.

Items are self‐rated from 1 to 10, yielding total scores from 34 to 340; higher scores indicate greater self‐reported DL. Because respondents rate how well each statement describes their own capability, the instrument measures perceived DL rather than objectively demonstrated performance in simulation or real‐world disaster response. Cronbach’s *α* was 0.986 in the present sample.

### 2.4. Statistical Analysis

Analyses were conducted using SPSS Version 26.0 and AMOS Version 24.0. Categorical variables were summarised as frequencies and percentages. Descriptive statistics were reported for both total scale scores and mean item scores. Mean item scores were used in the correlation and path analyses. In the analytic dataset, UWES responses were coded from 1 to 7 and were recoded to the instrument’s original 0–6 metric before scoring. Normality, outliers and multicollinearity were assessed before inferential analyses. Pearson correlations were calculated to examine the associations among POS, WE and DL. All tests were two‐tailed, with statistical significance set at *p* < 0.05.

A theory‐informed observed‐variable path analysis was conducted with POS specified as the predictor, WE as the intermediate variable and DL as the outcome. The adjusted model included gender, age, education, professional title, school‐based disaster education and on‐the‐job disaster training as covariates. Categorical covariates were dummy coded. Reference categories were male, age 26–30 years, bachelor’s degree, junior professional title, prior on‐the‐job disaster training = yes, and school‐based disaster education = no. Covariates were entered as predictors of both WE and DL. Full covariate estimates are reported in Supporting Table S1.

Indirect associations were estimated as the product of the POS‐to‐WE and WE‐to‐DL paths. Bias‐corrected 95% confidence intervals were generated using 5000 bootstrap resamples, and an indirect association was considered statistically significant when the confidence interval excluded zero. Direct, indirect and total associations were reported using unstandardised and standardised estimates where available. Potential common method variance was assessed using Harman’s single‐factor test [25, 26].

### 2.5. Ethical Considerations

The study was reviewed and approved by the Clinical Research Ethics Committee of the participating hospital. The ethics review was conducted on 31 July 2025, and the approval became effective on 1 August 2025 (approval no. PJ 2025‐06‐93), before participant recruitment and data collection, which were conducted between March and April 2026. All participants provided written informed consent before completing the questionnaire.

## 3. Results

### 3.1. Participant Characteristics

All 113 nurses in the defined reserve pool were included in the analysis. Ten participants (8.8%) were male, and 103 (91.2%) were female. Most were aged 31–40 years (*n* = 76, 67.3%), held a bachelor’s degree (*n* = 104, 92.0%) and had an intermediate professional title (*n* = 78, 69.0%). Ninety‐three nurses (82.3%) reported school‐based disaster education, and 111 (98.2%) reported on‐the‐job disaster training. A disaster‐response team had been established according to 109 participants (96.5%); four participants (3.5%) reported that they did not know, and none reported that a team had not been established.

The distributions show that the reserve population was relatively homogeneous in several respects, particularly gender, educational level, ethnicity and prior on‐the‐job disaster training. Participant characteristics are presented in Table 1.

**TABLE 1:** Participant characteristics (*N* = 113).

Variable	Category	*n* (%)
Gender	Male	10 (8.8)
	Female	103 (91.2)

Age	26–30 years	37 (32.7)
	31–40 years	76 (67.3)

Ethnicity	Han Chinese	111 (98.2)
	Ethnic minority	2 (1.8)

Education	Doctoral degree	0 (0.0)
	Master’s degree	7 (6.2)
	Bachelor’s degree	104 (92.0)
	Associate degree	2 (1.8)
	Technical secondary school	0 (0.0)

Marital status	Unmarried	20 (17.7)
	Married	93 (82.3)
	Cohabiting	0 (0.0)
	Divorced	0 (0.0)
	Separated	0 (0.0)
	Widowed	0 (0.0)

Professional title	Senior	0 (0.0)
	Intermediate	78 (69.0)
	Junior	35 (31.0)

Disaster‐response team established	Yes	109 (96.5)
	No	0 (0.0)
	Don’t know	4 (3.5)

On‐the‐job disaster training	Yes	111 (98.2)
	No	2 (1.8)

School‐based disaster education	Yes	93 (82.3)
	No	20 (17.7)

*Note:* Data are presented as *n* (%). Zero‐frequency response categories are retained for completeness. Percentages were calculated using *N* = 113; totals may differ from 100.0% because of rounding.

### 3.2. Descriptive Statistics and Correlations

The mean total scores were 56.75 (SD = 8.43) for POS, 77.31 (SD = 17.97) for WE and 288.34 (SD = 44.38) for DL. The corresponding mean item scores were 4.37 (SD = 0.65) for POS, 4.55 (SD = 1.06) for WE and 8.48 (SD = 1.31) for DL.

All three focal variables were positively correlated. POS was positively associated with WE (*r* = 0.588, *p* < 0.001) and DL (*r* = 0.540, *p* < 0.001), and WE was positively associated with DL (*r* = 0.565, *p* < 0.001). The descriptive statistics and correlation matrix are shown in Table 2.

**TABLE 2:** Descriptive statistics and Pearson correlations among study variables (*N* = 113).

Variable	Items	Range	M	SD	1	2	3
1. Perceived organisational support	13	1–5	4.37	0.65	1		
2. Work engagement	17	0–6	4.55	1.06	0.588^∗∗∗^	1	
3. Disaster literacy	34	1–10	8.48	1.31	0.540^∗∗∗^	0.565^∗∗∗^	1

*Note:* Mean item scores are reported.

^∗∗∗^ *p* < 0.001 (two‐tailed).

### 3.3. Theory‐Informed Path Analysis

In the fully adjusted model, POS was positively associated with WE (*B* = 0.929, SE = 0.125, *t* = 7.440, *p* < 0.001, *β* = 0.570, bias‐corrected 95% CI [0.694, 1.191]). WE was positively associated with DL after adjustment for POS and the covariates (*B* = 0.555, SE = 0.116, t = 4.780, *p* < 0.001, *β* = 0.449, bias‐corrected 95% CI [0.325, 0.815]). POS also retained a direct association with DL after WE and the covariates were included (*B* = 0.618, SE = 0.183, t = 3.380, *p* = 0.001, *β* = 0.307, bias‐corrected 95% CI [0.110, 1.069]). The adjusted path coefficients are presented in Table 3 and illustrated in Figure 1.

**TABLE 3:** Adjusted observed‐variable path coefficients (*N* = 113).

Outcome	Predictor (path)	*B*	SE	*t*	*p*	*β*	BC 95% CI
Work engagement	POS (*a*)	0.929	0.125	7.440	< 0.001	0.570	(0.694, 1.191)
Disaster literacy	WE (*b*)	0.555	0.116	4.780	< 0.001	0.449	(0.325, 0.815)
Disaster literacy	POS (*c*’)	0.618	0.183	3.380	0.001	0.307	(0.110, 1.069)

*Note:* Mean item scores were used. The model adjusted for gender, age, education, professional title, school‐based disaster education and on‐the‐job disaster training. Categorical covariates were dummy coded and entered as predictors of both WE and DL. Reference categories were male, age 26–30 years, bachelor’s degree, junior professional title, prior on‐the‐job disaster training = yes, and school‐based disaster education = no. Bias‐corrected 95% confidence intervals were based on 5000 case‐resampling bootstrap samples.

Abbreviations: DL, disaster literacy; POS, perceived organisational support; WE, work engagement.

**FIGURE 1:**
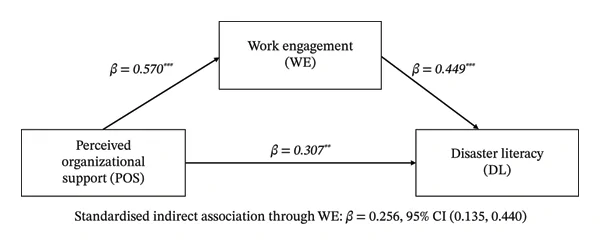
Standardised direct and indirect associations among perceived organisational support, work engagement and disaster literacy. *Note:* Standardised path coefficients are shown. The model adjusted for gender, age, education, professional title, school‐based disaster education and on‐the‐job disaster training; covariate paths are omitted for clarity. The 95% confidence interval for the indirect association was based on 5000 bias‐corrected bootstrap resamples. Arrows represent theory‐informed statistical paths and should not be interpreted as causal directions because the study was cross‐sectional. Abbreviations: DL = disaster literacy; POS = perceived organisational support; WE = work engagement. ^∗∗^ *p* < 0.01; ^∗∗∗^ *p* < 0.001.

The unstandardised indirect association through WE was 0.515 (SE = 0.151, bias‐corrected 95% CI [0.271, 0.890]), and the standardised indirect association was *β* = 0.256 (bias‐corrected 95% CI [0.135, 0.440]). The unstandardised direct association was 0.618 (bias‐corrected 95% CI [0.110, 1.069]), and the total model‐estimated association was 1.134 (SE = 0.163, bias‐corrected 95% CI [0.747, 1.459]). Full covariate estimates are provided in Supporting Table S1. The indirect and direct estimates represented approximately 45.5% and 54.5%, respectively, of the total model‐estimated association. These percentages describe a decomposition of the model coefficients, not the proportion of a causal effect mediated by WE. The bootstrap decomposition of the total model‐estimated association is presented in Table 4.

**TABLE 4:** Bootstrap decomposition of the total model‐estimated association (*N* = 113).

Association	*B*	SE	*z*/t	*p*	BC 95% CI	Coefficient decomposition (%)
Indirect (*a* × *b*)	0.515	0.151	3.407	< 0.001	(0.271, 0.890)	45.5
Direct (*c*’)	0.618	0.183	3.380	0.001	(0.110, 1.069)	54.5
Total (*c*)	1.134	0.163	6.974	< 0.001	(0.747, 1.459)	100.0

*Note:* Unstandardised estimates are reported. The standardised indirect association was *β* = 0.256 (bias‐corrected 95% CI [0.135, 0.440]). The percentage column is a mathematical decomposition of coefficients in this cross‐sectional model and must not be interpreted as the proportion of a causal effect mediated through work engagement.

### 3.4. Potential Common Method Variance

Harman’s single‐factor test showed that the first unrotated factor explained 54.52% of the total variance. This finding raises concern that common measurement conditions may have contributed to the observed associations among the scales. It does not quantify how much each correlation or path coefficient was affected and does not establish that the findings were entirely attributable to method bias.

Accordingly, the correlation and path results should be interpreted as associations among self‐reported measures collected at the same time. The common‐method finding was considered when interpreting both the magnitude and the practical implications of the estimates.

## 4. Discussion

### 4.1. Principal Findings

This study examined organisational and motivational correlates of DL in critical care reserve nurses. POS, WE and self‐reported DL were positively correlated. In the adjusted model, POS remained directly associated with DL, and the data were also consistent with an indirect association through WE. These findings accord with the JD–R model, in which job resources are associated with motivation and favourable work outcomes [14, 15]. These findings also suggest that preparedness among reserve nurses should be considered in relation to both technical competence and the organisational context in which nurses train and work. However, because the study was cross‐sectional and all focal variables were self‐reported, the findings should be interpreted as theory‐informed associations rather than evidence of causal mediation.

### 4.2. DL in Critical Care Reserve Nurses

The mean self‐reported DL score was 288.34 (SD = 44.38), higher than the mean of 255.62 reported in a national survey of Chinese nurses [11]. This difference may reflect the characteristics of the present sample: participants were members of a critical care reserve pool, worked in a tertiary hospital and had substantial previous exposure to disaster education or training. Because the survey preceded the 6 month training programme, the higher score cannot be attributed to that programme. Differences in sampling and reserve‐pool selection also limit direct comparisons with the national survey. Moreover, DL was self‐reported, and recent randomised evidence indicates that perceived confidence and assessed knowledge should be treated as related but distinct outcomes [27].

A high total DL score does not necessarily indicate uniformly high readiness across all domains. The ICN frameworks encompass preparation, communication, incident management, safety, assessment, intervention, recovery, and legal and ethical practice [4–6]. Reviews continue to identify gaps in practical skills and psychological preparedness [7, 8, 28], while multicentre and structural‐equation studies show that disaster competence and literacy vary across settings and are associated with social and psychological factors [29, 30]. Readiness assessment should therefore combine self‐reported literacy with objective simulation performance and domain‐specific feedback.

### 4.3. Organisational Support and DL

The positive association between POS and WE is consistent with previous studies of nurses, including those conducted in China and in intensive care settings [18, 19, 31]. POS reflects whether nurses believe that the organisation values their contribution and supports their well‐being [16, 17]. In the context of disaster preparedness, organisational support may take the form of access to training and information, clear role expectations, adequate resources, supportive supervision and explicit recognition of nurses’ reserve responsibilities.

POS also retained a direct association with DL after WE and the selected covariates were included. Organisational support may facilitate access to learning opportunities and encourage nurses to seek clarification, participate in exercises and discuss errors or uncertainty. Visible investment by managers may also signal that preparedness is an organisational priority [21]. Because these management practices were not measured, the observed association warrants further investigation but does not demonstrate that increasing POS alone would improve DL.

### 4.4. Indirect Association Through WE

WE was positively associated with DL, and the bootstrap confidence interval for the indirect association excluded zero. This finding is consistent with research identifying WE as part of pathways linking organisational resources with nurses’ occupational outcomes [32]. Engaged nurses may make more active use of preparedness resources by seeking current information, practising unfamiliar procedures, participating in simulations and reflecting on performance. Within the JD–R framework, POS can be viewed as a job resource associated with WE, a motivational state that may encourage nurses to use preparedness‐related resources more actively. This interpretation is theoretical; the cross‐sectional data do not establish temporal order or a causal mechanism.

Nevertheless, the variables were measured concurrently. Greater perceived DL could itself strengthen professional confidence and engagement, and unit culture or prior emergency experience may influence all three variables. The indirect and direct estimates represented approximately 45.5% and 54.5%, respectively, of the total model‐estimated association. These values describe only the mathematical decomposition of coefficients in the specified cross‐sectional model and should not be interpreted as proportions of a causal effect mediated through WE.

### 4.5. Common Method and Interpretive Caution

Harman’s single‐factor test showed that the first unrotated factor explained 54.52% of the total variance, suggesting that common method variance cannot be excluded. Because POS, WE and DL were measured by self‐report at the same time, shared response tendencies may have inflated the observed associations. Harman’s test cannot quantify the extent of this inflation [25, 26], but the result supports cautious interpretation of coefficient magnitude. Future studies should use repeated measurements, multiple data sources and objective preparedness outcomes.

### 4.6. Implications for Nursing Management

Although the cross‐sectional findings do not demonstrate that increasing organisational support or WE improves DL, they provide a rationale for prospectively evaluating management strategies that address these factors in critical care reserve nurses.

The findings suggest that reserve‐workforce development should combine technical training with organisational and motivational support. Nurse managers can facilitate preparedness by providing protected training time, access to current guidance, clear reserve roles and transparent activation procedures. Psychologically safe debriefing may help nurses discuss uncertainty and performance gaps, while authentic roles, familiar clinical environments and skilled facilitation may strengthen learning transfer during in situ simulation [33].

Preparedness activities should also promote sustained participation through scenario‐based simulation, interprofessional drills, case review and structured reflection. Practical, clinically situated training may improve both confidence and assessed knowledge beyond mandatory online education [27]. Feedback should be timely and linked to functional, interactive and critical dimensions of DL.

These strategies should be evaluated rather than assumed to be effective. Hospitals could monitor participation, completion of actions arising from feedback and retention in the reserve pool, alongside objective drill performance and repeated assessments of WE and DL. Structured tools developed for evaluating disaster drills may support this process [34]. Because needs may differ across hospitals and levels of prior experience, management strategies should be adapted to local workforce and organisational conditions.

### 4.7. Limitations

Several limitations should be considered. First, the study included 113 nurses from a single critical care reserve pool at one tertiary hospital. The sample was also highly homogeneous and demographically imbalanced, particularly in gender, educational level, ethnicity and prior on‐the‐job disaster training. The findings therefore should not be generalised to primary‐hospital nurses, nonreserve nursing staff or critical care reserve nurses in other hospitals or regions without external validation. The modest sample size also limited the precision of covariate estimates, particularly for sparsely represented categories.

Second, the cross‐sectional design did not establish temporal ordering and could not exclude reverse or reciprocal relationships. Because data were collected before the six month critical care training programme, the study cannot evaluate the effectiveness of that programme.

Third, all focal variables were self‐reported at one time point, creating risks of social desirability and common method bias. Harman’s single‐factor test provides only a preliminary assessment of common method variance, and a common‐latent‐factor sensitivity analysis was not conducted; residual common method variance therefore could not be quantified.

Fourth, the analyses used composite scale scores and assessed perceived DL rather than demonstrated performance. They therefore did not address domain‐specific pathways or actual performance during deployment. Future multicentre longitudinal studies should combine repeated self‐report measures with objective simulation scores, behavioural observation and supervisor or peer assessment.

## 5. Conclusion

Among critical care reserve nurses in one tertiary hospital, perceived organisational support, WE and self‐reported DL were positively associated. The adjusted model was consistent with both a direct association between perceived organisational support and self‐reported DL and an indirect association through WE. This pattern supports the relevance of organisational resources and motivation to disaster‐workforce development, but it does not establish a causal mechanism.

For nurse managers, the findings justify prospective evaluation of preparedness strategies that combine technical training with protected learning time, clear communication, psychologically safe debriefing and visible support. The magnitude of these associations should be interpreted cautiously because the study was single‐centre and cross‐sectional, with all key variables measured by self‐report at the same time. Multicentre longitudinal studies using objective performance indicators are needed before these management strategies can be considered evidence‐based interventions.

## Author Contributions

Chang Shu and Zhijun Shu contributed equally to study design, data collection, data analysis and manuscript drafting. Ziyan Yu and Lilan Wang contributed to data collection and manuscript revision. Bingbing Hu contributed to study implementation and substantive manuscript revision. Shaohua Hu supervised the study, contributed to interpretation of results and critically revised the manuscript.

## Funding

This work was supported by the Provincial Quality Project of the Education Department of Anhui Province (Grant no. 2024jyxm0715), the Young Research Project of the Chinese Nursing Association (Grant no. ZHKYQ202402), and the Scientific Research Project of the Education Department of Anhui Province (Grant no. 2025AHGXZK20012).

## Disclosure

All authors read and approved the final manuscript.

## Conflicts of Interest

The authors declare no conflicts of interest.

## Supporting Information

Additional supporting information can be found online in the Supporting Information section.

## Supporting information


**Supporting Information** Supporting Table S1. Full covariate estimates from the adjusted observed‐variable path analysis.

## Data Availability

The de‐identified data that support the findings of this study are available from the corresponding author upon reasonable request, subject to approval by the participating institution and applicable ethical restrictions.

## References

[1] Brown L. M. , Haun J. N. , and Peterson L. , A Proposed Disaster Literacy Model, Disaster Medicine and Public Health Preparedness. (2014) 8, no. 3, 267–275, 10.1017/dmp.2014.43. 24992944

[2] Zhang D. , Development of Disaster Literacy Model of Clinical Nurses,Construction of Influencing Factors Model and Research on Intervention Strategies (Doctoral Dissertation), 2022, China Medical University, Shenyang, China, 10.27652/d.cnki.gzyku.2022.000309.

[3] Zhang D. , Zhang L. Y. , Zhang X. , and Zhu X. F. , Improve Disaster Literacy in Nurses: A Qualitative Descriptive Study, BMC Nursing. (2024) 23, no. 1, 10.1186/s12912-024-02381-2. PMC11462798 39379933

[4] Dorsey D. M. , World Health Organization Regional Office for the Western Pacific, and International Council of Nurses, ICN Framework of Disaster Nursing Competencies. (2009) WHO Regional Office for the Western Pacific and International Council of Nurses, Manila.

[5] International Council of Nurses , Core Competencies in Disaster Nursing:Version 2.0, 2019, Author, Geneva, Switzerland.

[6] International Council of Nurses , Core Competencies in Disaster Nursing:Competencies for Nurses Involved in Emergency Medical Teams(Level III), 2022, Author, Geneva, Switzerland.

[7] Feng J. , Zhang C. , Fang S. , Zhao R. , Wang H. , and Li D. , Influencing Factors of Nurses’Disaster Preparedness:A Systematic Review and meta-analysis, BMC Public Health. (2025) 25, no. 1, 10.1186/s12889-025-23981-w. PMC12326711 40770316

[8] Labrague L. J. , Hammad K. , Gloe D. S. et al., Disaster Preparedness Among Nurses:A Systematic Review of Literature, International Nursing Review. (2018) 65, no. 1, 41–53, 10.1111/inr.12369. 28295314

[9] Luo Y. , Yan H. , Tang Y. et al., Levels of Nurse Disaster Preparedness:A Systematic Review and meta-analysis, Nurse Education in Practice. (2025) 85, 10.1016/j.nepr.2025.104372. 40267854

[10] Li Z. and Sheng Y. , An Investigation of Emergency Nurses’Preparedness for Disasters in China, Chinese Journal of Nursing. (2014) 49, 699–703, 10.3761/j.issn.0254-1769.2014.06.015.

[11] Zhang D. and Zhang L. , Disaster Literacy Status and Influencing Factors: a National Survey of 107,997 Nurses, Journal of Sichuan University (Medical Science Edition). (2023) 54, no. 4, 824–830, 10.12182/20230760101. PMC10442629 37545081

[12] Liu H. , Zhao H. , Zeng Y. et al., Development and Application of a Training System for Nurses’Comprehensive Emergency Response Competency, Chinese Journal of Nursing. (2019) 54, 1136–1141, 10.3761/j.issn.0254-1769.2019.08.003.

[13] Setyawati A. D. and Lu Y. Y. , Theoretical-Based Training Programs in Disaster Nursing: A Systematic Review, Disaster Medicine and Public Health Preparedness. (2025) 19, 10.1017/dmp.2025.10142. 40660775

[14] Bakker A. B. and Demerouti E. , Job Demands–Resources Theory: Taking Stock and Looking Forward, Journal of Occupational Health Psychology. (2017) 22, no. 3, 273–285, 10.1037/ocp0000056. 27732008

[15] Demerouti E. , Bakker A. B. , Nachreiner F. , and Schaufeli W. B. , The Job Demands-Resources Model of Burnout, Journal of Applied Psychology. (2001) 86, no. 3, 499–512, 10.1037/0021-9010.86.3.499. 11419809

[16] Eisenberger R. , Armeli S. , Rexwinkel B. , Lynch P. D. , and Rhoades L. , Reciprocation of Perceived Organizational Support, Journal of Applied Psychology. (2001) 86, no. 1, 42–51, 10.1037/0021-9010.86.1.42. 11302232

[17] Rhoades L. and Eisenberger R. , Perceived Organizational Support: A Review of the Literature, Journal of Applied Psychology. (2002) 87, no. 4, 698–714, 10.1037/0021-9010.87.4.698. 12184574

[18] Gupta V. , Agarwal U. A. , and Khatri N. , The Relationships Between Perceived Organizational Support,Affective Commitment,Psychological Contract Breach,Organizational Citizenship Behaviour and Work Engagement, Journal of Advanced Nursing. (2016) 72, no. 11, 2806–2817, 10.1111/jan.13043. 27293180

[19] Gao X. , Zhou Y. , Xu X. , Yuan R. , Zheng Y. , and Yun R. , Relationships Between Organizational Support,Emotional Intelligence and Work Engagement Among Chinese Nurses: A Correlation Study, Nursing Open. (2024) 11, no. 10, 10.1002/nop2.70034. PMC11474886 39404068

[20] Wu M. , Wang W. , Chen W. , and Qiu H. , Mediating Effect of Psychological Capital Between Perceived Organizational Support and Work Engagement in Chinese Nurses: A Systematic Review and Meta-Analytic Structural Equation Modeling, Psychology, Health & Medicine. (2026) 1–20, 10.1080/13548506.2026.2628982. 41679748

[21] Connelly B. L. , Certo S. T. , Ireland R. D. , and Reutzel C. R. , Signaling Theory: A Review and Assessment, Journal of Management. (2011) 37, no. 1, 39–67, 10.1177/0149206310388419.

[22] Schaufeli W. B. , Salanova M. , González-Romá V. , and Bakker A. B. , The Measurement of Engagement and Burnout: A Two Sample Confirmatory Factor Analytic Approach, Journal of Happiness Studies. (2002) 3, no. 1, 71–92, 10.1023/A:1015630930326.

[23] Zuo H.-M. and Yang H. , A Study on Relationship Between Organizational Support Emotion and Organizational Commitment of Nurses, Chinese Nursing Research. (2009) 23, 1341–1343.

[24] Zhang Y.-W. and Gan Y.-Q. , The Chinese Version of Utrecht Work Engagement Scale: An Examination of Reliability and Validity, Chinese Journal of Clinical Psychology. (2005) 13, 268–270, 10.16128/j.cnki.1005-3611.2005.03.005.

[25] Podsakoff P. M. , MacKenzie S. B. , Lee J.-Y. , and Podsakoff N. P. , Common Method Biases in Behavioral Research: A Critical Review of the Literature and Recommended Remedies, Journal of Applied Psychology. (2003) 88, no. 5, 879–903, 10.1037/0021-9010.88.5.879. 14516251

[26] Xiong H.-X. , Zhang J. , Ye B.-J. , Zheng X. , and Sun P.-Z. , Common Method Variance Effects and the Models of Statistical Approaches for Controlling It, Advances in Psychological Science. (2012) 20, no. 5, 757–769, 10.3724/SP.J.1042.2012.00757.

[27] Durbridge N. , Hall E. , Sikic M. , Weber A. , Thompson J. , and O’Reilly G. , Evaluating the Difference in Emergency Nurses’Disaster Response Self-Reported Confidence and Assessed Knowledge Scores: A Randomized Controlled Trial, Disaster Medicine and Public Health Preparedness. (2025) 19, 10.1017/dmp.2025.10076. 40545801

[28] Said N. B. and Chiang V. C. L. , The Knowledge,Skill Competencies,And Psychological Preparedness of Nurses for Disasters: A Systematic Review, International Emergency Nursing. (2020) 48, 10.1016/j.ienj.2019.100806. 31685363

[29] Yang Y. , Zhang Y. , Liu Y. et al., The Associated Factors of Disaster Literacy Among Nurses in China: A Structure Equation Modelling Study, BMC Nursing. (2024) 23, no. 1, 10.1186/s12912-024-02486-8. PMC11590267 39587594

[30] Zhu D. , Li Y. , Chen Q. et al., Status and Influencing Factors of Clinical Nurses’ Disaster Nursing Competency: A Multicenter Cross-Sectional Study, BMC Nursing. (2025) 24, no. 1, 10.1186/s12912-025-03026-8. PMC11971766 40188344

[31] Li H. , Wang J. , Dai H. et al., Organizational Support and Work Engagement in General Intensive Care Unit Nurses: The Double-Edged Sword Effect of Emotional Labor, Journal of Nursing Management. (2026) 2026, no. 1, 10.1155/jonm/4743882. PMC12961163 41799944

[32] Ren Y. , Li G. , Pu D. et al., The Relationship Between Perceived Organizational Support and Burnout in Newly Graduated Nurses From Southwest China: The Chain Mediating Roles of Psychological Capital and Work Engagement, BMC Nursing. (2024) 23, no. 1, 10.1186/s12912-024-02386-x. PMC11459715 39379880

[33] Krarup K. B. , McKenzie A. C. , Al-Masri A. , and Abildgren L. , Authentic Roles Matter: How in Situ Simulation Influences Fidelity,Psychological Safety, And Learning Transfer, Advances in Simulation. (2026) 11, no. 1, 10.1186/s41077-026-00449-4. PMC13591749 42260636

[34] Alanazi M. A. , Assessing the Impact of Disaster Drills on Emergency Nurses: Development and Validation of the Disaster Drills Effectiveness Questionnaire (DDEQ), BMC Nursing. (2025) 24, no. 1, 10.1186/s12912-025-03364-7. PMC12211761 40597145

